# Effusive constrictive pericarditis and an anterior mediastinal mass: a case report

**DOI:** 10.1093/ehjcr/ytag020

**Published:** 2026-01-24

**Authors:** Madiha Kiyani, Waleed R Chaudhry, Yakubu Bene-Alhasan, Shaikh B Iqbal

**Affiliations:** Department of Medicine, MedStar Health–Georgetown University (Baltimore), 201 E University Pkwy, Baltimore, MD 21218, USA; Services Institute of Medical Sciences, G8QM+JWR, Jail Rd, Shadman 1 Shadman, Lahore 54000, Pakistan; Department of Medicine, MedStar Health–Georgetown University (Baltimore), 201 E University Pkwy, Baltimore, MD 21218, USA; Department of Medicine, MedStar Union Memorial Hospital, 201 E University Pkwy, Baltimore, MD 21218, USA; Department of Cardiology, Section of Vascular Medicine, Massachusetts General Hospital, 55 Fruit St, Boston, MA 02114, USA

**Keywords:** Effusive-constrictive pericarditis, ECP, Anterior mediastinal mass, B-cell lymphoma, Case report

## Abstract

**Background:**

The presence of effusive-constrictive pericarditis (ECP) in the context of malignancies, especially large B-cell lymphoma is rare. Recognizing this presentation is crucial due to its life-threatening nature, as the combination of ECP and cardiac tamponade can cause severe haemodynamic instability in patients.

**Case summary:**

A 22-year-old man presented to the emergency department with a 1-month history of progressively worsening shortness of breath, night sweats, anorexia, 15 pounds weight loss, and palpitations. Laboratory findings revealed elevated white blood cell count, lactate dehydrogenase, D-dimer, lactic acid, and erythrocyte sedimentation rate. Computed tomography scan confirmed a large anterior mediastinal mass, a large pericardial effusion, and moderate-sized pleural effusions bilaterally. Echocardiography confirmed the large pericardial effusion, prompting urgent pericardiocentesis, which yielded 450 cc fluid. Next morning, he redeveloped chest discomfort and tachycardia. Repeating echocardiography redemonstrated moderate-sized pericardial effusion with extensive loculation, fibrinous material, early diastolic collapse, and respiratory variation in mitral inflow, pointing towards ECP with possible tamponade. The patient was started on colchicine and transferred to a tertiary care hospital for further management. Follow-up echocardiograms over there kept showing partly organized focal pockets of pericardial effusion with a small area of echogenic material but no haemodynamic instability. Biopsy, taken from the anterior mediastinal mass, confirmed the diagnosis of primary mediastinal large B-cell lymphoma, and he was started on of rituximab, cyclophosphamide, doxorubicin, vincristine, and prednisone chemotherapy.

**Conclusion:**

Effusive-constrictive pericarditis is an uncommon but critical manifestation of primary mediastinal large B-cell lymphoma. Combination of large pericardial effusion with constrictive features and persistent non-compliant pericardium with elevated intracardiac pressures after pericardiocentesis are its hallmarks.

Learning points

- Effusive-constrictive pericarditis is characterized by persistent pericardial constriction post-fluid drainage and is often underdiagnosed. It rarely presents with lymphoma.
- Echocardiography is essential for diagnosis in the absence of cardiac magnetic resonanc imaging (MRI) and biopsy for fibrinous material/loculations, respiratory variation in mitral inflow velocity and expiratory diastolic flow reversal in the hepatic vein.

## Introduction

Effusive-constrictive pericarditis (ECP) is characterized by the simultaneous presence of a haemodynamically significant pericardial effusion and reduced pericardial compliance, which leads to impaired cardiac filling.^1^ Lymphomas account for ∼5%–15% of pericardial effusions. Patients who present with moderate to large effusions, cardiac tamponade, or ECP are more likely to be diagnosed with an underlying malignancy.^2^ Early recognition and differentiation of pericardial disease presentations are essential for guiding diagnostic workup and management.^3^ We present an interesting case of a 22-year-old healthy man with ECP complicated by tamponade due to an anterior mediastinal mass.

## Summary figure

**Figure:**
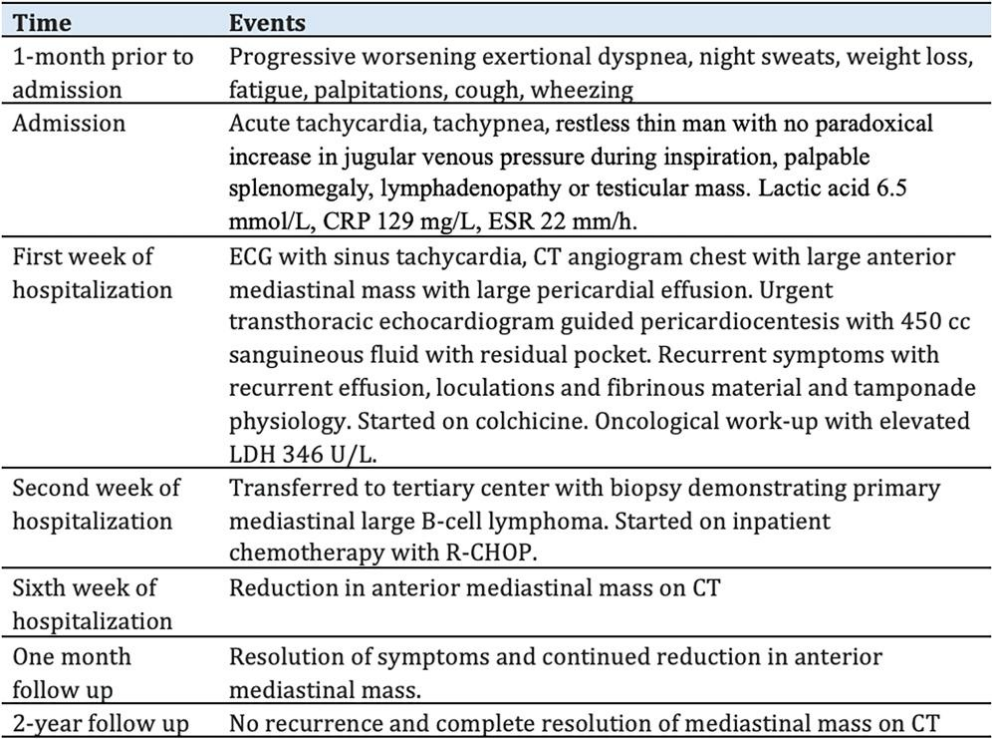


## Case presentation

A 22-year-old man presented to the emergency department with a 1-month history of progressively worsening shortness of breath. His past medical history was notable for suspected uncontrolled asthma and eczema. His shortness of breath limited his daily activities and was associated with intermittent productive cough, palpitations, and dizziness. He later endorsed for 2 weeks prior to presentation that he also had fatigue, decreased appetite, night sweats, and 15 pounds of weight. While in the ED, he was afebrile (36.8°C), tachycardic (149 beats per minute), tachypnoeic (38 breaths per minute), and normotensive (128/86 mmHg) and saturating 98% on room air. Physical exam was notable for a restless thin man with tachycardia with no paradoxical increase in jugular venous pressure during inspiration, palpable splenomegaly, lymphadenopathy, or testicular mass.

**Figure 1:**
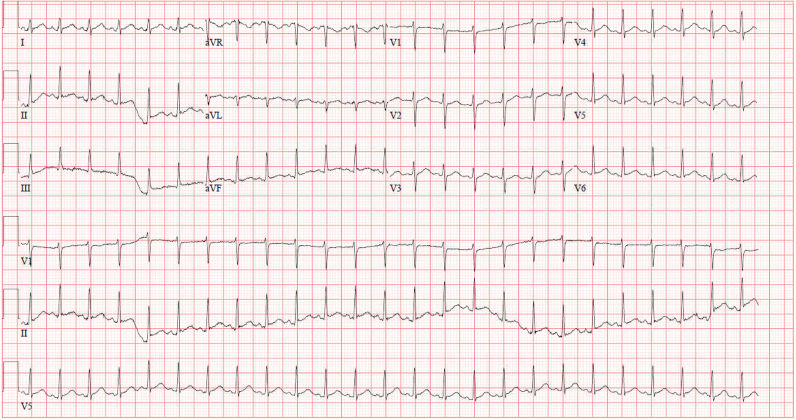
Twelve-lead electrocardiogram showing sinus tachycardia.

**Figure 2:**
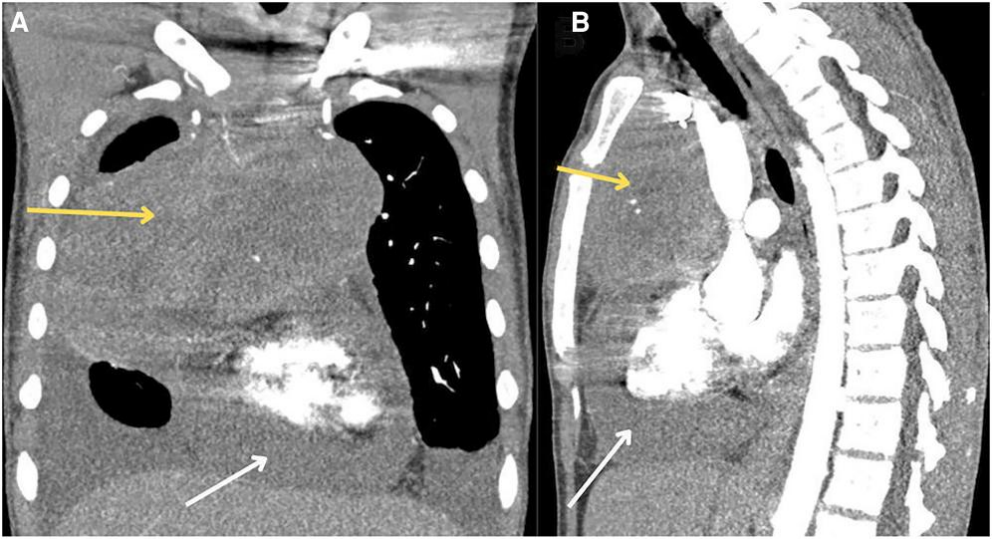
(*A*) Computed tomography chest coronal view demonstrating large anterior mass (yellow arrow), large pericardial effusion (white arrow), and bilateral pleural effusions. (*B*) Computed tomography chest sagittal view demonstrating large anterior mass (yellow arrow) and large pericardial effusion (white arrow).

**Figure 3:**
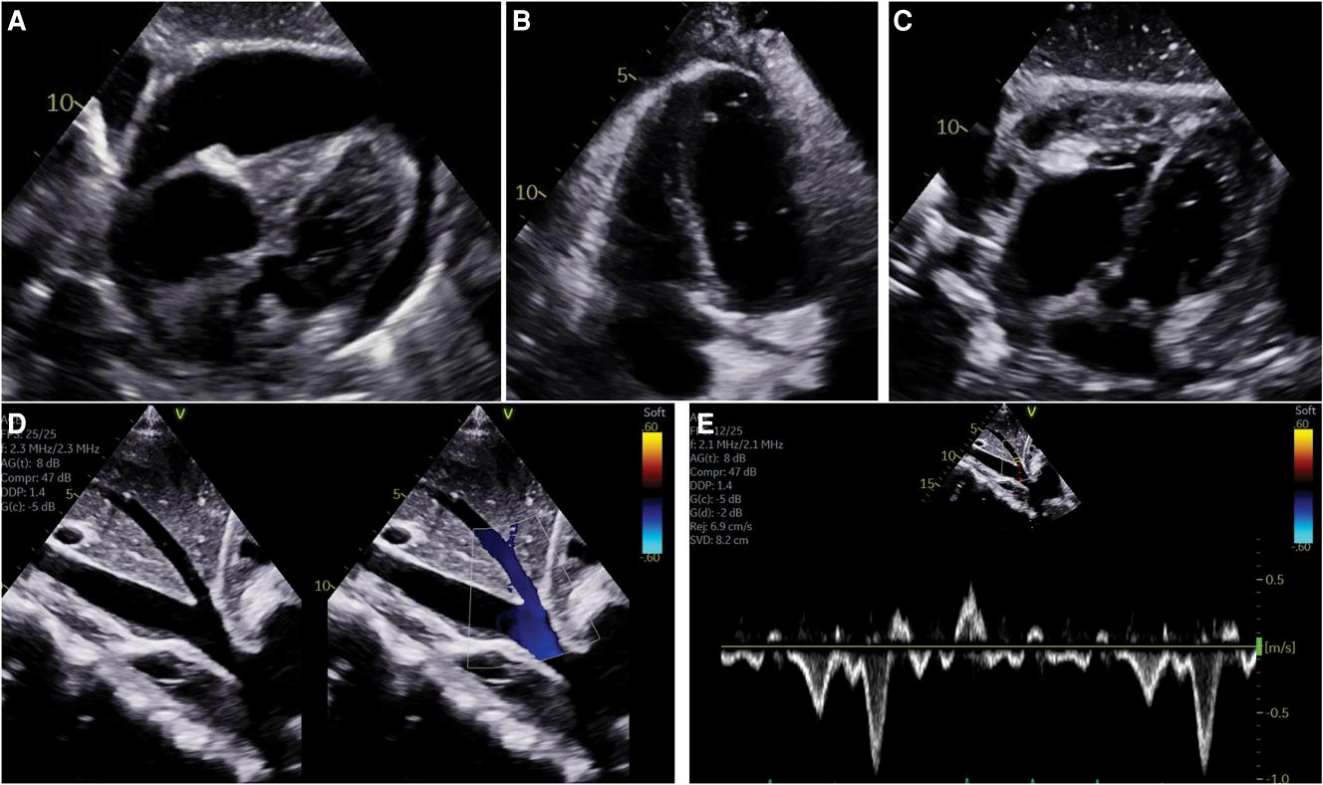
(*A*) Four-chamber view demonstrating large pericardial effusion with right ventricular collapse. (*B*) Four-chamber view, post-pericardiocentesis demonstrating resolution of pericardial effusion. (*C*) Five-chamber view demonstrating focal pockets of pericardial fluid with fibrinous strands. (*D–E*) Post-pericardiocentesis with expiratory diastolic flow reversal of hepatic vein.

Initial laboratory investigations revealed an elevated white blood cell count (13.0 k/μL; reference range, 4.0–10.3 k/μL), a markedly elevated D-dimer (9.81 mcg/mL; normal high < 0.5 mcg/mL), elevated lactic acid (6.5 mmol/L; reference range, 0.7–2.0 mmol/L), elevated C-reactive protein (129.0 mg/L: reference range, 0.0–3.0 mg/L), and elevated erythrocyte sedimentation rate (ESR) (22 mm/h; reference range, 0.0–15.0 mm/h). Electrocardiogram demonstrated sinus tachycardia (*Figure 1*). Chest X-ray demonstrated bilateral pleural effusions with abnormal density in the right hemithorax. Computed tomography (CT) angiogram of the chest showed a large anterior mediastinal mass measuring 15.3 × 8.3 × 1.6 cm, large pericardial effusion without pericardial calcifications, and moderate loculated pleural effusions bilaterally (*Figure 2*). An urgent limited echocardiogram confirmed a large pericardial effusion with signs of tamponade (*Figure 3A*). An urgent pericardiocentesis was completed with drainage of 450 cc sanguineous pericardial fluid and notable residual fluid pocket inferior to the right ventricle (*Figure 3B*).

A few hours later, the patient began to have chest discomfort with tachypnoea and tachycardia. He had minimal pericardial fluid output. Transthoracic echocardiogram with Doppler revealed moderate-sized pericardial effusion with extensive loculations and fibrinous material mostly surrounding the right and left ventricles, the inferolateral pericardial space and the apex, early diastolic collapse and invagination of the right ventricle, respiratory variation of mitral valve inflow, and expiratory diastolic flow reversal in the hepatic vein (*Figure 3D-E, Video 1*). The inferior vena cava (IVC) was dilated and non-collapsible and exhibited interventricular dependence. These findings were consistent with ECP with tamponade physiology. Compared to prior post-pericardiocentesis images, there was a modest increase in pericardial effusion and new fibrinous material in the pericardial space. Oncological work-up included serum alpha-fetoprotein (AFP) and beta-human chorionic gonadotropin (β-HCG) levels, which were within normal limits, and lactate dehydrogenase (LDH), which was elevated (346 U/L; reference range, 87–241 U/L), and cytology was negative for malignant cells.

**Figure 4:**
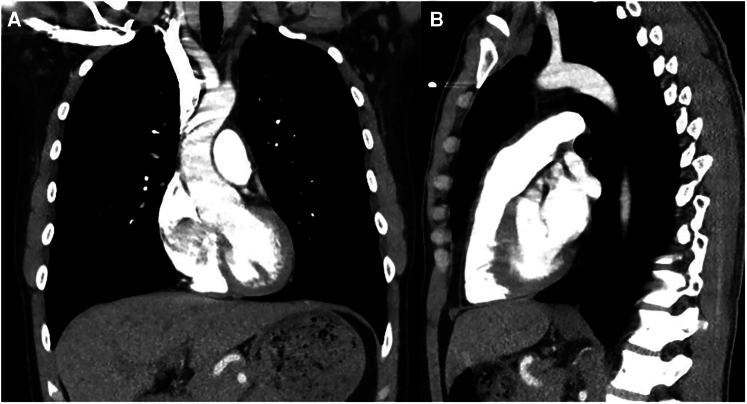
(*A*) Computed tomography chest coronal view demonstrating resolution of anterior mass. (*B*) Computed tomography chest sagittal view demonstrating resolution of anterior mass.

The patient was transferred to a tertiary care hospital for further management of anterior mediastinal mass. He underwent a biopsy of the anterior mediastinal mass, which confirmed primary mediastinal large B-cell lymphoma. He was started on inpatient chemotherapy and completed six cycles of rituximab, cyclophosphamide, doxorubicin, vincristine, and prednisone (R-CHOP). At outpatient follow-up at 4 months, the patient was asymptomatic with serial imaging demonstrating reduction in mass. Computed tomography angiogram chest at 2-year follow-up demonstrated complete resolution (*Figure 4*).

## Discussion

Effusive-constrictive pericarditis is characterized by haemodynamically significant pericardial effusion and decreased pericardial compliance. It is most clearly demonstrated in patients who continue to exhibit elevated intracardiac pressures even after pericardiocentesis.^3^ Common aetiologies of ECP include tuberculosis, viral infection, medications, radiation, cardiac surgery, autoimmune disease, and malignancy post-procedural complications, radiation therapy, drug-induced connective tissue diseases, post-myocardial infarction (Dressler syndrome), malignancy, trauma, and uraemia.^3,4^ Our case presented with an anterior mediastinal mass due to large B-cell lymphoma and cardiac tamponade. Lymphomas are associated with B-symptoms that consist of systemic symptoms of fever, night sweats, and weight loss and typically associated with localized lymphadenopathy.^5^ However, mediastinal involvement presenting with pericardial effusion resulting in ECP is an unusual manifestation. Only a few cases of lymphoma causing ECP have been documented.^6–8^

Traditionally, the diagnosis of ECP is based on invasive haemodynamic assessment. However, certain echocardiographic features, especially those noted after pericardiocentesis, can help in identifying this condition.^9^ In our patient, the initial signs of cardiac tamponade included a large pericardial effusion, diastolic collapse, respiratory inflow variation, and a dilated IVC. These findings persisted even after pericardiocentesis. Despite draining 450 cc of fluid, the patient experienced recurrent symptoms, including chest discomfort and tachycardia along with minimal drain output. The lack of resolution in respiratory variation of mitral inflow following fluid removal is an important diagnostic clue.^10^ The persistence of restrictive forces on the heart despite fluid relief indicates presence of ECP.^11^ Another interesting aspect of our case was negative cytology, which makes us think that the effusion could have resulted from a paraneoplastic inflammatory response without direct tumour infiltration, possible lymphatic obstruction, or early disease or low tumour burden in the pericardium rather than from a malignant cells shedding into the pericardial fluid.^2^ B-cell lymphomas can sometimes present with indolent behaviour, leading to a lower presence of malignant cells in the effusion. The fluid can be predominantly inflammatory with lymphocytes and may not contain enough atypical cells to warrant a positive cytology.^2^ It is important to note that ECP is often underdiagnosed due to subtle echocardiographic findings after pericardiocentesis.^12^ Advanced imaging modalities, such as cardiac MRI and CT, may be necessary to identify pericardial thickening or calcification associated with ECP.^12^

After drainage, our patient was treated conservatively with colchicine while being monitored closely for pulsus paradoxus and haemodynamic decompensation. The stabilization of the patient’s symptoms suggests that conservative management with colchicine was effective in controlling the constrictive physiology, providing a rare insight into medical management of effusive-constrictive cases. Pericardiectomy is usually reserved for chronic constrictive cases, which do not respond to initial therapy.^13^ The standard chemotherapy for patients with diffuse large B-cell lymphoma is treatment with R-CHOP. Radiation may be added for bulky masses without improvement in chemotherapy. However, progressive disease after R-CHOP chemotherapy should undergo repeat biopsy to confirm diagnosis and undergo combination salvage therapy.^5^

## Patient’s perspective

The patient was satisfied with his relief of symptoms, the care he received between both hospital systems, and the sustained response to his chemotherapy.

## Conclusion

Patients with large anterior mediastinal masses with pericardial effusions require urgent evaluation with echocardiogram and multidisciplinary approach for successful treatment.

## Lead author biography


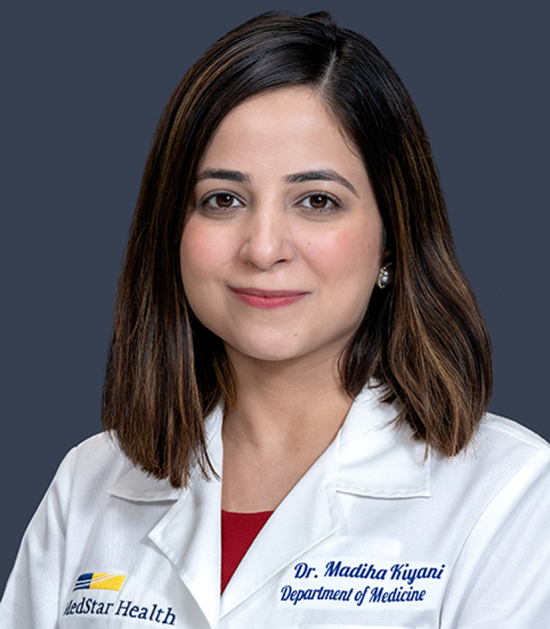


Madiha Kiyani is an internal medicine resident at MedStar Health–Georgetown University, Baltimore, Maryland, USA. She graduated with her MBBS degree from Faisalabad Medical University, Faisalabad, Pakistan. Following residency training, she is interested in pursuing a fellowship in cardiovascular disease.

## Supplementary Material

ytag020_Supplementary_Data

## Data Availability

No new data were generated or analysed in support of this research.

## Notes

**Consent:** Patient’s consent was obtained in compliance with the COPE guidelines.
